# Innovative Models of Care for Hospitals of the Future

**DOI:** 10.34172/ijhpm.2024.7861

**Published:** 2024-02-18

**Authors:** Robyn Clay-Williams, Peter Hibbert, Graeme Loy, Jeffrey Braithwaite

**Affiliations:** ^1^Australian Institute of Health Innovation, Faculty of Medicine, Health and Human Sciences, Macquarie University, Sydney, NSW, Australia.; ^2^Western Sydney Local Health District, Sydney, NSW, Australia.

**Keywords:** Hospital Design, Integrated Care, Qualitative Research, Acute Care

## Abstract

New ways of providing acute care outside of traditional hospital building complexes, such as virtual care or hospital in the home, are becoming more common. Despite this, many hospitals are still conceived as "bricks and mortar" centralised constructions, and few health service infrastructure organisations meet intensively with consumers or clinicians prior to conceptualising hospital design. Our study sought to understand the needs and expectation of community members and healthcare providers, and co-design innovative models of acute care to inform development of a new metropolitan hospital in Australia. Our study used a three-step approach, consisting of academic and grey literature reviews; a demographic analysis of the hospital catchment population; and a series of 20 workshops and 6 supplementary interviews with community members and local healthcare providers. We found that care should be tailored to the healthcare needs and expectations of each consumer, with consumers cared for in the community where possible and safe. We propose an innovative model of care for hospitals of the future, consisting of fully integrated acute care underpinned by appropriate digital architecture to deliver care that is community focussed. It is vital that new hospitals build in sufficient adaptability to leverage future innovation and meet the needs of growing and changing communities.

## Background

 Hospital design is evolving in response to many architectural, social, technological and financial factors, but especially increasing consumer demands and lessons learned from the COVID-19 pandemic.^1,2^ New ways of providing acute care outside of traditional hospital building complexes, such as virtual care or hospital in the home, are becoming more common.^3,4^ When establishing new hospitals, health jurisdictions are looking to more distributed models that promote alternative patient care pathways, and enable greater choice and flexibility for both consumers and healthcare providers.

 Despite recognition of the need to develop patient-centred models of care, few health service infrastructure organisations meet intensively with consumers prior to conceptualising hospital design.^5,6^ Where consumers or providers are consulted as part of the design process, this is typically in the later stages where the building structure has been locked in and the remaining questions are around décor and organisation of interior spaces.^5,7^ In our study, we employed a co-design approach with a health infrastructure organisation, health service district leaders, clinicians and consumers at the conceptual stage, to inform the development of a new, community-focused hospital in a fast-growing metropolitan health district in Australia.

## Methods

 To develop strategies for providing acute care for the new hospital, we employed a three-step approach (Figure). First, we sought to understand world’s best practice for innovative ways of providing acute care through a review of the grey and academic literature.^8^ Second, we assessed the characteristics of the community that the hospital would support through a demographic analysis of the projected hospital catchment population. Third, we elicited community and healthcare provider expectations for the new hospital through a series of workshops and interviews.^9^ Then we applied a multi-level model variant^9^ of the triangulation method to integrate findings from the three steps and arrive at broad guidance for designing a hospital to meet the future needs of the health district.

**Figure:**
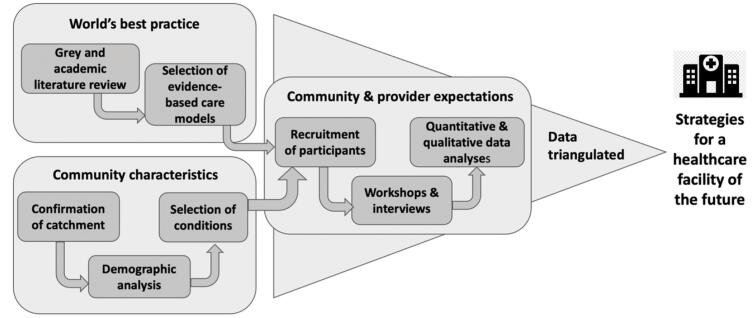
Project Overview.

## Results

###  World’s Best Practice

 To identify innovative approaches to delivering acute care in Australia and internationally, we screened 2858 grey literature articles published between 2010 and 2020, and reviewed 82 that met inclusion criteria. Data were thematically analysed and six innovative approaches to delivering care were identified, with consumer focussed care as an overall theme: (1) ambulatory care and diagnostics, (2) hospital in the home, (3) integrated care, (4) telehealth and virtual care, (5) digital hospitals, and (6) specialised hospitals with population specific care units. We then searched the peer-reviewed academic literature over the same period to identify evidence on the implementation and effectiveness of these seven models. We identified 10 676 articles, from which 61 reviews and seven primary papers met inclusion criteria. The reviews synthesised findings from 1154 primary studies undertaken across 19 countries.

 In the literature, delivery of consumer-focused care was a common aim across all models. Focussing care on consumer needs and preferences was consistently associated with positive outcomes, including improved patient health outcomes and experiences, and better relationships between consumers and providers.^8^ The remaining six models had common strengths and weaknesses across health conditions, but their effectiveness was heavily dependent on context and choosing an appropriate mode of implementation. A strong theme in the literature on innovation involved moving care from treatment delivered in inpatient settings towards care delivered in outpatient settings, in the community or in the consumer’s home.^8^

 Overall, the solutions that produced the best outcomes blended two or more models, and supported care delivery with appropriate and well-integrated technology. Successful combinations included blending specialist hospitals with integrated care, and blending virtual care with hospital in the home.^8^

###  Community Characteristics

 At almost 500 km^2^, the hospital catchment was large and consisted of 49 suburbs.^10^ The population in 2019 was approximately 300 000 residents, and was expected to grow by nearly 30% over the next 10 years.^11^ A high proportion of young families lived in the area; the most recent Census reported the largest age groups were adults aged 35-44 years and school children aged 5-9 years.^11^ The area was culturally diverse with over one third of the population born outside Australia, and 37% speaking a language other than English at home.^12^ Common health conditions identified within the population were kidney disease, heart disease, and type II diabetes.^12^

###  Community and Provider Consultations

 Twenty workshops and six interviews were conducted to elicit the acute care needs and treatment expectations of 158 participants (community members [n = 84] and healthcare providers [n = 74]). Illustrative patient vignettes were developed for each of the six models or approaches to care that were identified in the literature (ambulatory care, digital hospitals, hospital in the home, integrated care, virtual care, and specialist hospitals) and used to guide discussion.

 Participants represented a diverse range in age, background, ethnicity and profession from across the hospital catchment. Provider participants ranged from district hospital employees to community health providers and general practitioners (GPs). All workshops were conducted online, as there were COVID-19 restrictions in place at the time; while participants reported diverse levels of digital literacy, it is possible that community members with low levels of digital literacy were unable to participate. One workshop was delivered in Mandarin with the aid of interpreters; however, 36% the consumers who attended the main workshops also reported fluency in a language other than English, in line with the population’s profile.

 Transcripts of the interviews and scribed notes from the workshop were aggregated into two files (consumers and providers) and thematically analysed. The findings identified benefits, drawbacks and enablers for each of the models of care and their implementation, and participant views on the types of health condition that were best suited to each.^13-16^

 The study found no single model that worked best for all consumers, all providers or all conditions. Rather, it was perceived as important to tailor the care delivery to meet the healthcare needs and expectations of each consumer, with providers formulating their scope of practice in accordance with evidence-based treatment guidelines and availability of health service resources. The optimum general approach was to care for consumers in the community where possible, only bringing them into an acute care facility if warranted due to the severity or complexity of their health condition, or their self- or clinician-assessed inability to cope with lower acuity care. To be successful, this arrangement would require greater integration of services than is currently in place; particularly integration of primary, acute and specialist care, and implementation of fully integrated communication technology (ICT) to support streamlined transition between services. Access to acute care should always be available locally, provided that sufficient access to local care to meet community expectations can be provided through virtual or hospital in the home modes. Acute care needs, including escalation to in-hospital services when needed, could be adequately addressed through efficient and accessible transport and ICT systems, and delivered via existing or expanded health district services.

## Discussion

###  Strategies for Designing a Healthcare Facility of the Future

 We found that early consultation with consumer and provider stakeholders, and integrating what was learned with analysis of community characteristics and evidence-based international best practice, provided a robust basis for hospital conceptual design. In addition to meeting community needs and expectations, a key message from the study was that a hospital must build in sufficient adaptability to leverage future innovation and meet the needs of a growing and changing community. Flexible patient pathways that allow for personal choice and individualised medicine must also be incorporated into the design. Resourcing should not be overlooked: clinical staffing needs are critical, as is sufficient access to hospital beds, outpatients, and community-based services.

 Provided that infrastructure and care delivery design issues are resolved, a key innovation for the new hospital will be successful implementation of the blended innovative models of care, rather than the individual models themselves. Many of the models have already been adopted (or partially adopted) in different forms in many jurisdictions,^8^ but successful and sustainable implementation at scale of innovative models and approaches has been elusive. For example, integrated care consisting of connected community and primary care with tertiary hospital care has yet to be attained across developed health systems despite its documented, obvious advantages.^17^ Specific barriers and enablers for design and implementation of the new healthcare facility were identified in our study. For example, enablers include providing easy access to services and facilities (eg, public transport, parking); facilitating consumer choice and support; providing sufficient staffing and resources to deliver safe and high quality care; and supporting integrated care models with effective multidisciplinary team communication strategies; and developing safety plans for escalating care when treatment does not go as expected. Vital to adoption of the conceptual model is the appropriate digital architecture to underpin it, integrating hospital infrastructure, including ICT services with GPs and community.

 Utilising an evidence-based approach to implementation, guided by an Implementation Science framework,^18,19^ such as the Consolidated Framework for Implementation Research,^20^ can be vital for success. Frameworks such as the Consolidated Framework for Implementation Research have proven to be effective for implementing large scale, complex interventions in healthcare, by guiding adaptation of the intervention to meet specific health system needs, and addressing barriers and enablers to safeguard optimum uptake. From the literature,^21,22^ general barriers and enablers for implementation of the innovative models of care include the need to facilitate change through modifying attitudes and behaviour at the individual, team and organisational levels; the need to address financial and other resourcing constraints; and the need to enact structural, policy and legal level change.^23^ To ensure any new model of care maintains a strong community-focus in the face of competing priorities, it is important to include consumer representatives from the hospital catchment in the implementation team.

###  How the Health District Used the Research Findings

 To ensure the successful translation of this research into a practical and deliverable facility design a comprehensive consultation model with the clinical leaders from our partner health district occurred. This was delivered through three “think tank” sessions. Here, the research framework was presented to the senior clinicians, a vision for a best practice centre for innovation in healthcare was described, and permission given to blue sky thinking for how clinical services can be delivered.

 Integral to the concept of the hospital of the future was the requirement for the design to map the patient journey from prevention to intervention to wellness, in a one health system model that straddles primary care, community care and acute care delivery. At its core is the concept that a patient should not need to navigate multiple systems, rather the systems present as a seamless continuity of care. To deliver that, we incorporated a Community Care model,^24^ originally developed to address COVID-19, where the hospital partnered with on site GPs, an independent urgent care service, an extensive outpatient model that included prehabilitation, and a targeted range of acute services designed around the health needs of the local community.

 This innovative model enables healthcare interventions to be streamed to the most appropriate care providers and commences from the first point of patient contact with the whole health system, wherever that may be. From paramedic access to multiple models, the prehab services support reduced demand for acute care; this model will provide seamless access to the most appropriate care provider in the best location for the patient.

## Ethical issues

 Ethics approval for conducting the study was obtained from the Western Sydney Local Health District Human Research Ethics Committee (Approval Number: 2021/PID01000). Written consent was obtained from all participants.

## Competing interests

 Graeme Loy is an employee of the Local Health District where the new hospital will be built. The remaining authors have no conflicts to declare.

## Funding

 The research was funded by a grant from Health Infrastructure, New South Wales, Australia [Grant number: HI20314]. The funder was involved in developing the objective of the research, but had no role in conducting the study or in determining the content of the manuscript.
